# Crystal structure of penta­kis­(ethyl­enedi­amine-κ^2^ *N*,*N*′)lanthanum(III) trichloride–ethylene­diamine–dichloromethane (1/1/1)

**DOI:** 10.1107/S1600536814023289

**Published:** 2014-10-29

**Authors:** Hope T. Sartain, Richard J. Staples, Shannon M. Biros

**Affiliations:** a Department of Chemistry, Grand Valley State University, Allendale, MI 49401, USA; b Center for Crystallographic Research, Department of Chemistry, Michigan State University, East Lansing, MI 48824, USA

**Keywords:** crystal structure, ethyl­enedi­amine, rare earth element

## Abstract

We report here the crystal structure of a ten-coordinate lanthanum(III) metal coordinated by five bidentate ethyl­enedi­amine ligands, [La(C_2_H_8_N_2_)_5_]Cl_3_·C_2_H_8_N_2_·CH_2_Cl_2_. One free ethyl­enedi­amine mol­ecule and three Cl^−^ anions are also located in the asymmetric unit. The overall structure is held together by an extensive hydrogen-bonding network between the Cl^−^ anions and the NH groups on the metal-bound ethyl­enedi­amine ligands. The free ethyl­enedi­amine mol­ecule is held in an ordered position by additional hydrogen bonds involving both the chlorides and –NH groups on the metal-bound ligands. One highly disordered mol­ecule of di­chloro­methane is located on an inversion center; however, all attempts to model this disorder were unsuccessful. The electron density in this space was removed using the BYPASS procedure [van der Sluis & Spek (1990 ▶). *Acta Cryst.* A**46**, 194–201].

## Abstract 2

The crystal structure of the title compound consists of a ten-coordinate lanthanum(III) cation chelated by five ethyl­enedi­amine ligands. This complex co-crystallized with one mol­ecule of ethyl­enedi­amine and three chloride anions.

## Chemical context   

The coordination chemistry of rare earth elements has impact in the areas of nuclear power, light-emitting diodes, medical imaging agents, and fluorescent sensors. The geometry of this ten-coordinate lanthanum(III) structure is of inter­est to researchers developing high denticity ligands for lanthanides and actinides.
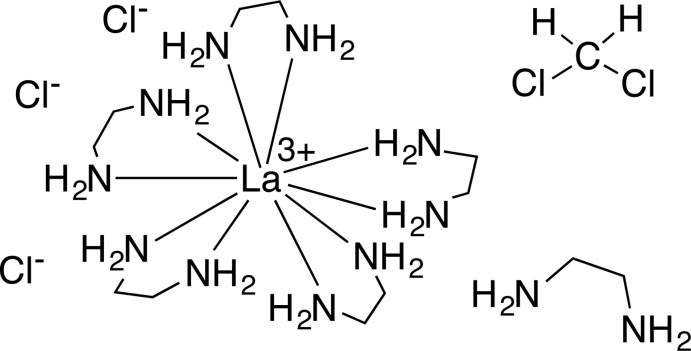


## Structural commentary   

**Figure 1:**
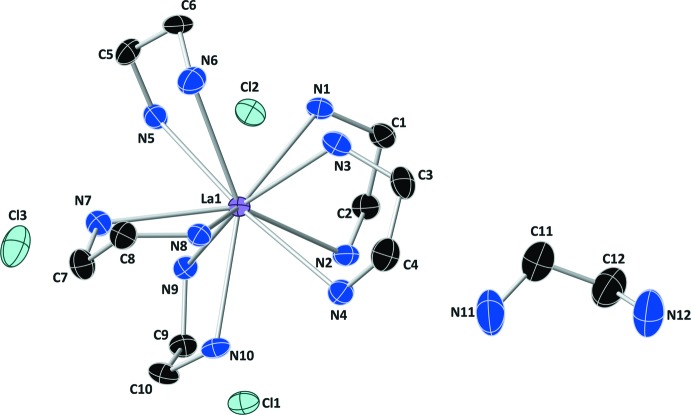
The asymmetric unit of the title crystal structure, showing displacement ellipsoids at the 50% probability level. H atoms have been omitted for clarity. Color codes: black C, blue N, purple La, light blue Cl.

**Figure 2:**
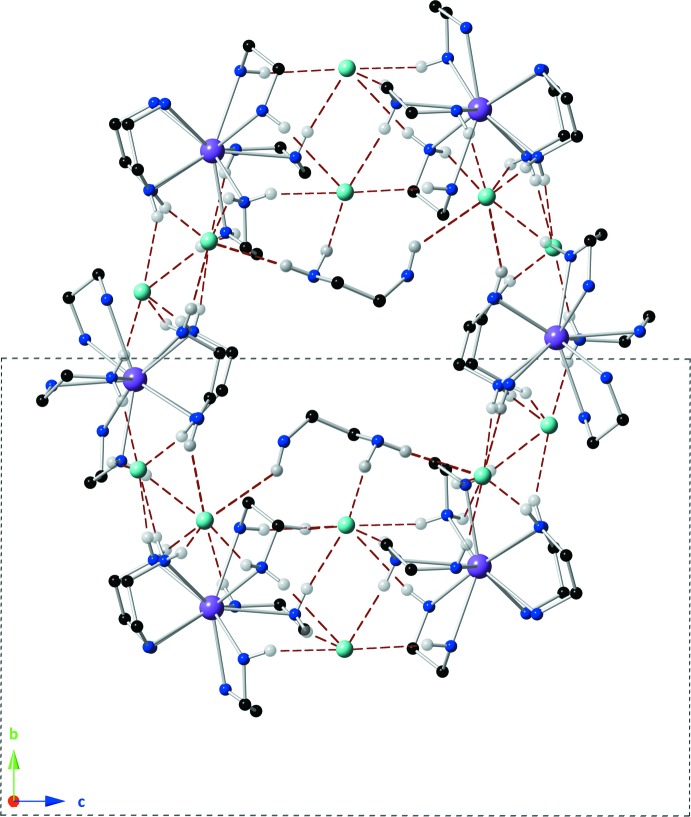
The hydrogen-bonding network surrounding one chair-shaped void, viewed down the *a* axis. The center of the void lies on an inversion center. H atoms not involved in a hydrogen bond have been omitted for clarity. Hydrogen bonds are shown as red dashed lines. Color codes as in Fig. 1 ▶.

The asymmetric unit of the title compound contains one La^III^ ion chelated by five ethyl­enedi­amine mol­ecules, one unbound ethyl­enedi­amine mol­ecule, and three chloride ions (Fig. 1 ▶). The coordination geometry of the La^3+^ ion resembles a distorted bicapped square anti­prism [range of La—N bond lengths = 2.715 (3)–2.876 (3) Å]. Inter­estingly, all three Cl^−^ ions and the free ethyl­enedi­amine mol­ecule are involved in an extensive hydrogen-bonding network that acts to rigidify the three-dimensional structure within the crystal lattice (see Figs. 2 ▶ and 3 ▶, and Table 1 ▶).

Each asymmetric unit contains one small void that lies on an inversion center (see the *Supra­molecular features* and *Refinement* sections for more discussion on the contents and treatment of this void).

## Supra­molecular features   

Six La^3+^-containing complex cations and twelve Cl^−^ anions are arranged in a rough hexa­gon in the *bc* plane (Fig. 2 ▶). The center of this hexa­gon contains two free ethyl­enedi­amine mol­ecules involved in extensive hydrogen bonding with the Cl^−^ ions and bound –NH groups of the lanthanum complex. The relatively non-polar portion of the free ethyl­enedi­amine mol­ecules face the inter­ior of the hexa­gon, which creates a void that resembles the shape of the chair conformation of cyclo­hexane. There are two of these voids per unit cell (see *Refinement* section) each located about an inversion center and likely containing one highly disordered di­chloro­methane mol­ecule.

**Figure 3:**
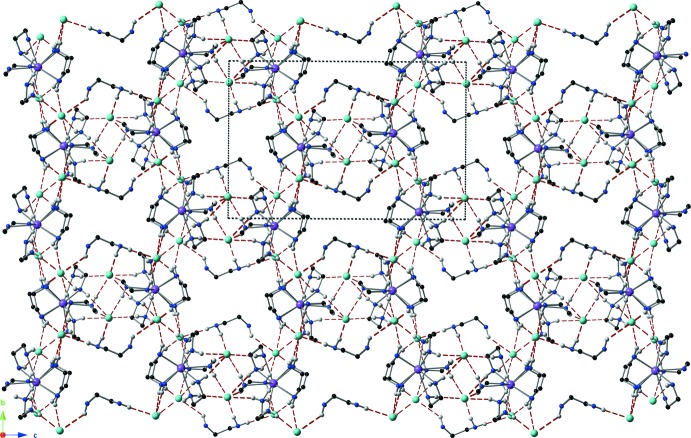
The extended hydrogen-bonding network forming a honeycomb-like network, viewed down the *a* axis. H atoms not involved in a hydrogen bond have been omitted for clarity. Hydrogen bonds are shown as red dashed lines. Color codes as in Fig. 1 ▶.

A view of the packing down the *a* axis (Fig. 3 ▶) reveals that the lanthanum complexes are arranged into a honeycomb-like lattice. Each side of the lanthanum complex supra­molecular hexa­gon is shared with a neighboring hexa­gon and held together with extensive hydrogen-bonding inter­actions (Table 1 ▶).

## Database survey   

Related structures involving a lanthanum(III) ion coordinated by three or more ethyl­enedi­amine ligands have been reported by Jia *et al.* (2005 ▶, 2006 ▶), Feng *et al.* (2009 ▶) and Chen *et al.* (2009 ▶).

## Synthesis and crystallization   

Crystals suitable for X-ray diffraction studies were serendip­itously grown from the vapor diffusion of a 3:1 ethyl­enedi­amine–di­chloro­methane solution into a saturated solution of the lanthanum(III)–ligand complex previously reported by our group (Sartain *et al.*, 2014 ▶) in aceto­nitrile.

## Refinement   

Crystal data, data collection and structure refinement details are summarized in Table 2 ▶. H atoms were placed in calculated positions and constrained to ride on their parent atoms, with *U*_iso_(H) = 1.2*U*_eq_(C,N) for methyl­ene and amino groups. In the free ethyl­enedi­amine mol­ecule, N—H distances were restrained to 0.9 Å using DFIX instructions in *SHELXL* (Sheldrick, 2008 ▶). If these hydrogens were left unrestrained, the result was bond lengths that were outside accepted values.

There are two small void spaces, each located on an inversion center, per unit cell. The coordinates of the inversion centers are (0, ½, 0) and (½, 0, ½). Attempts to model a disordered di­chloro­methane mol­ecule in this void were unsuccessful. The intensity contribution of the disordered solvent mol­ecules was removed by the BYPASS procedure (van der Sluis & Spek, 1990 ▶), as implemented in *OLEX2* (Dolomanov *et al.*, 2009 ▶, 2014 ▶). The size of the void was calculated to be 153.6 Å^3^, containing approximately 35 electrons.

## Supplementary Material

Crystal structure: contains datablock(s) I. DOI: 10.1107/S1600536814023289/pk2532sup1.cif

Structure factors: contains datablock(s) I. DOI: 10.1107/S1600536814023289/pk2532Isup2.hkl

CCDC reference: 1030544

Additional supporting information:  crystallographic information; 3D view; checkCIF report

## Figures and Tables

**Table 1:** Hydrogen-bond geometry (, )

*D*H*A*	*D*H	H*A*	*D* *A*	*D*H*A*
N1H1*C*Cl2^i^	0.91	2.53	3.405(3)	160
N2H2*C*N11	0.91	2.40	3.284(5)	164
N2H2*D*Cl2^ii^	0.91	2.72	3.417(3)	134
N3H3*D*Cl2	0.91	2.59	3.497(3)	176
N4H4*D*N11	0.91	2.49	3.267(5)	144
N5H5*C*Cl2^i^	0.91	2.74	3.573(3)	153
N7H7*C*Cl3^iii^	0.91	2.54	3.376(3)	154
N7H7*D*Cl3	0.91	2.63	3.470(3)	154
N8H8*D*Cl2	0.91	2.40	3.292(3)	168
N10H10*C*Cl2^ii^	0.91	2.57	3.445(3)	161
N11H11*D*Cl2^ii^	1.00(5)	2.83(5)	3.642(5)	139(4)

Symmetry codes: (i) 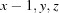; (ii) 
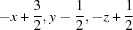
; (iii) 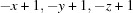.

**Table 2:** Experimental details

Crystal data	
Chemical formula	[La(C_2_H_8_N_2_)_5_]Cl_3_C_2_H_8_N_2_CH_2_Cl_2_
*M*_r_	690.78
Crystal system, space group	Monoclinic, *P*2_1_/*n*
Temperature (K)	173
*a*, *b*, *c* ()	8.8070(7), 14.6530(12), 22.1110(18)
()	92.1560(9)
*V* (^3^)	2851.4(4)
*Z*	4
Radiation type	Mo *K*
(mm^1^)	1.99
Crystal size (mm)	0.26 0.20 0.08

Data collection	
Diffractometer	Bruker APEXII CCD
Absorption correction	Multi-scan (*SADABS*; Bruker, 2012 ▶)
*T*_min_, *T*_max_	0.641, 0.745
No. of measured, independent and observed [*I* > 2(*I*)] reflections	24978, 5609, 4502
*R*_int_	0.056
(sin /)_max_ (^1^)	0.618

Refinement	
*R*[*F* ^2^ > 2(*F* ^2^)], *wR*(*F* ^2^), *S*	0.033, 0.077, 1.07
No. of reflections	5609
No. of parameters	265
No. of restraints	2
H-atom treatment	H atoms treated by a mixture of independent and constrained refinement
_max_, _min_ (e ^3^)	0.80, 0.48

Computer programs: *APEX2* and *SAINT* (Bruker, 2012 ▶), *SHELXS2013* and *SHELXL97* (Sheldrick, 2008 ▶), *CrystalMaker* (Palmer, 2007 ▶) and *OLEX2* (Dolomanov *et al.*, 2009 ▶, 2014 ▶).

## supplementary crystallographic information

### Crystal data

**Table:**

[La(C_2_H_8_N_2_)_5_]Cl_3_·C_2_H_8_N_2_·CH_2_Cl_2_	*F*(000) = 1248
*M**_r_* = 690.78	*D*_x_ = 1.609 Mg m^−^^3^
Monoclinic, *P*2_1_/*n*	Mo *K*α radiation, λ = 0.71073 Å
*a* = 8.8070 (7) Å	Cell parameters from 9898 reflections
*b* = 14.6530 (12) Å	θ = 2.3–26.0°
*c* = 22.1110 (18) Å	µ = 1.99 mm^−^^1^
β = 92.1560 (9)°	*T* = 173 K
*V* = 2851.4 (4) Å^3^	Block, colourless
*Z* = 4	0.26 × 0.20 × 0.08 mm

### Data collection

**Table:**

Bruker APEXII CCD diffractometer	4502 reflections with *I* > 2σ(*I*)
φ and ω scans	*R*_int_ = 0.056
Absorption correction: multi-scan (*SADABS*; Bruker, 2012)	θ_max_ = 26.0°, θ_min_ = 1.7°
*T*_min_ = 0.641, *T*_max_ = 0.745	*h* = −10→10
24978 measured reflections	*k* = −18→18
5609 independent reflections	*l* = −27→27

### Refinement

**Table:**

Refinement on *F*^2^	Primary atom site location: structure-invariant direct methods
Least-squares matrix: full	Hydrogen site location: mixed
*R*[*F*^2^ > 2σ(*F*^2^)] = 0.033	H atoms treated by a mixture of independent and constrained refinement
*wR*(*F*^2^) = 0.077	*w* = 1/[σ^2^(*F*_o_^2^) + (0.0267*P*)^2^ + 1.7999*P*] where *P* = (*F*_o_^2^ + 2*F*_c_^2^)/3
*S* = 1.07	(Δ/σ)_max_ = 0.001
5609 reflections	Δρ_max_ = 0.80 e Å^−^^3^
265 parameters	Δρ_min_ = −0.48 e Å^−^^3^
2 restraints	

### Special details

**Table:**

Experimental. Absorption correction: SADABS-2012/1 (Bruker, 2012) was used for absorption correction. wR2(int) was 0.0852 before and 0.0598 after correction. The Ratio of minimum to maximum transmission is 0.8603.
Geometry. All e.s.d.'s (except the e.s.d. in the dihedral angle between two l.s. planes) are estimated using the full covariance matrix. The cell e.s.d.'s are taken into account individually in the estimation of e.s.d.'s in distances, angles and torsion angles; correlations between e.s.d.'s in cell parameters are only used when they are defined by crystal symmetry. An approximate (isotropic) treatment of cell e.s.d.'s is used for estimating e.s.d.'s involving l.s. planes.

### Fractional atomic coordinates and isotropic or equivalent isotropic displacement parameters (Å^2^)

**Table:**

	*x*	*y*	*z*	*U*_iso_*/*U*_eq_	
C1	0.2628 (4)	0.5116 (2)	0.18320 (17)	0.0262 (9)	
H1A	0.3319	0.5161	0.1491	0.031*	
H1B	0.1660	0.5421	0.1712	0.031*	
C2	0.2336 (4)	0.4129 (3)	0.19737 (18)	0.0266 (9)	
H2A	0.1632	0.4083	0.2311	0.032*	
H2B	0.1860	0.3823	0.1615	0.032*	
C3	0.7194 (5)	0.5131 (3)	0.16495 (17)	0.0310 (9)	
H3A	0.7760	0.5549	0.1388	0.037*	
H3B	0.6242	0.4949	0.1429	0.037*	
C4	0.8134 (5)	0.4305 (3)	0.17969 (19)	0.0339 (10)	
H4A	0.8398	0.3991	0.1419	0.041*	
H4B	0.9090	0.4490	0.2013	0.041*	
C5	0.3650 (5)	0.6338 (3)	0.40189 (17)	0.0305 (9)	
H5A	0.4208	0.6271	0.4414	0.037*	
H5B	0.2716	0.6694	0.4085	0.037*	
C6	0.4623 (5)	0.6844 (3)	0.35888 (19)	0.0344 (10)	
H6A	0.4039	0.6961	0.3205	0.041*	
H6B	0.4929	0.7439	0.3766	0.041*	
C7	0.7721 (4)	0.4076 (3)	0.44040 (17)	0.0294 (9)	
H7A	0.7795	0.3422	0.4296	0.035*	
H7B	0.7965	0.4139	0.4843	0.035*	
C8	0.8836 (4)	0.4622 (3)	0.40492 (17)	0.0269 (9)	
H8A	0.8835	0.5264	0.4187	0.032*	
H8B	0.9873	0.4374	0.4121	0.032*	
C9	0.3576 (4)	0.2426 (2)	0.35430 (17)	0.0237 (8)	
H9A	0.3327	0.2148	0.3143	0.028*	
H9B	0.2949	0.2125	0.3847	0.028*	
C10	0.5227 (4)	0.2286 (2)	0.37051 (18)	0.0257 (9)	
H10A	0.5466	0.2532	0.4115	0.031*	
H10B	0.5468	0.1626	0.3707	0.031*	
C11	0.4553 (7)	0.3735 (4)	0.0489 (2)	0.0655 (16)	
H11A	0.5073	0.4325	0.0428	0.079*	
H11B	0.3597	0.3868	0.0694	0.079*	
C12	0.4146 (6)	0.3342 (4)	−0.0121 (2)	0.0622 (15)	
H12A	0.3609	0.2755	−0.0072	0.075*	
H12B	0.3452	0.3765	−0.0346	0.075*	
N1	0.3317 (3)	0.55679 (19)	0.23696 (13)	0.0219 (7)	
H1C	0.2558	0.5739	0.2613	0.026*	
H1D	0.3785	0.6086	0.2247	0.026*	
N2	0.3778 (3)	0.3682 (2)	0.21431 (14)	0.0252 (7)	
H2C	0.4356	0.3653	0.1811	0.030*	
H2D	0.3583	0.3100	0.2260	0.030*	
N3	0.6840 (3)	0.5592 (2)	0.22174 (14)	0.0256 (7)	
H3C	0.6236	0.6081	0.2126	0.031*	
H3D	0.7722	0.5810	0.2390	0.031*	
N4	0.7274 (3)	0.3678 (2)	0.21809 (14)	0.0260 (7)	
H4C	0.7945	0.3301	0.2380	0.031*	
H4D	0.6663	0.3325	0.1936	0.031*	
N5	0.3228 (3)	0.5422 (2)	0.37834 (14)	0.0256 (7)	
H5C	0.2345	0.5475	0.3559	0.031*	
H5D	0.3041	0.5054	0.4104	0.031*	
N6	0.5999 (4)	0.6293 (2)	0.34667 (15)	0.0329 (8)	
H6C	0.6588	0.6260	0.3813	0.039*	
H6D	0.6545	0.6590	0.3186	0.039*	
N7	0.6166 (3)	0.4406 (2)	0.42665 (13)	0.0245 (7)	
H7C	0.6057	0.4964	0.4440	0.029*	
H7D	0.5497	0.4020	0.4439	0.029*	
N8	0.8420 (3)	0.45799 (19)	0.33983 (13)	0.0212 (7)	
H8C	0.8860	0.4073	0.3245	0.025*	
H8D	0.8844	0.5071	0.3218	0.025*	
N9	0.3234 (3)	0.34180 (19)	0.35234 (13)	0.0209 (7)	
H9C	0.3059	0.3606	0.3907	0.025*	
H9D	0.2357	0.3499	0.3299	0.025*	
N10	0.6137 (3)	0.27590 (19)	0.32585 (14)	0.0226 (7)	
H10C	0.6034	0.2455	0.2900	0.027*	
H10D	0.7132	0.2725	0.3383	0.027*	
N11	0.5512 (6)	0.3182 (4)	0.08964 (19)	0.0620 (13)	
H11C	0.637 (6)	0.307 (4)	0.067 (2)	0.074*	
H11D	0.517 (6)	0.255 (4)	0.100 (2)	0.074*	
N12	0.5498 (5)	0.3195 (4)	−0.04637 (19)	0.0626 (13)	
H12C	0.517 (6)	0.301 (4)	−0.0835 (13)	0.075*	
H12D	0.599 (5)	0.271 (3)	−0.028 (2)	0.075*	
Cl2	1.01159 (10)	0.64537 (6)	0.29560 (4)	0.0277 (2)	
Cl3	0.29715 (13)	0.36410 (9)	0.49867 (5)	0.0470 (3)	
La1	0.54253 (2)	0.45443 (2)	0.30593 (2)	0.01558 (7)	
Cl1	0.99179 (10)	0.25647 (6)	0.30115 (4)	0.0258 (2)	

### Atomic displacement parameters (Å^2^)

**Table:**

	*U*^11^	*U*^22^	*U*^33^	*U*^12^	*U*^13^	*U*^23^
C1	0.027 (2)	0.023 (2)	0.028 (2)	0.0032 (17)	−0.0073 (17)	0.0034 (17)
C2	0.023 (2)	0.026 (2)	0.031 (2)	−0.0002 (17)	−0.0042 (17)	−0.0012 (17)
C3	0.029 (2)	0.040 (2)	0.024 (2)	−0.0064 (19)	0.0041 (18)	0.0083 (18)
C4	0.026 (2)	0.043 (3)	0.032 (2)	0.0007 (19)	0.0099 (19)	−0.0028 (19)
C5	0.036 (2)	0.031 (2)	0.025 (2)	0.0080 (19)	0.0063 (18)	−0.0065 (18)
C6	0.051 (3)	0.0153 (19)	0.036 (2)	0.0070 (19)	−0.004 (2)	−0.0030 (18)
C7	0.028 (2)	0.034 (2)	0.026 (2)	−0.0024 (18)	−0.0077 (17)	0.0045 (18)
C8	0.023 (2)	0.028 (2)	0.029 (2)	−0.0052 (17)	−0.0054 (17)	−0.0026 (17)
C9	0.022 (2)	0.0191 (19)	0.030 (2)	−0.0055 (16)	0.0024 (16)	0.0000 (16)
C10	0.023 (2)	0.0169 (18)	0.037 (2)	−0.0005 (16)	−0.0039 (17)	0.0050 (17)
C11	0.093 (4)	0.062 (4)	0.041 (3)	0.004 (3)	0.005 (3)	−0.009 (3)
C12	0.069 (4)	0.071 (4)	0.047 (3)	0.010 (3)	0.005 (3)	−0.014 (3)
N1	0.0183 (15)	0.0187 (16)	0.0285 (17)	0.0004 (13)	−0.0002 (13)	0.0018 (13)
N2	0.0290 (18)	0.0188 (16)	0.0277 (18)	0.0046 (14)	−0.0014 (14)	−0.0011 (14)
N3	0.0191 (16)	0.0244 (18)	0.0331 (19)	0.0015 (13)	−0.0003 (14)	0.0061 (14)
N4	0.0235 (17)	0.0281 (18)	0.0262 (17)	0.0037 (14)	−0.0023 (14)	−0.0019 (15)
N5	0.0274 (17)	0.0266 (17)	0.0230 (17)	0.0043 (14)	0.0027 (14)	0.0037 (14)
N6	0.041 (2)	0.0261 (18)	0.0322 (19)	−0.0063 (16)	0.0094 (16)	−0.0028 (15)
N7	0.0230 (16)	0.0242 (17)	0.0261 (18)	−0.0044 (14)	−0.0022 (14)	0.0012 (14)
N8	0.0227 (16)	0.0177 (15)	0.0233 (16)	−0.0037 (13)	0.0027 (13)	0.0012 (13)
N9	0.0175 (15)	0.0222 (16)	0.0231 (16)	0.0008 (13)	0.0001 (13)	−0.0019 (13)
N10	0.0181 (16)	0.0167 (15)	0.0328 (18)	0.0022 (13)	−0.0012 (14)	−0.0048 (14)
N11	0.079 (4)	0.076 (3)	0.031 (2)	−0.011 (3)	0.003 (2)	0.004 (2)
N12	0.058 (3)	0.093 (4)	0.037 (3)	0.007 (3)	0.004 (2)	−0.005 (3)
Cl2	0.0252 (5)	0.0238 (5)	0.0344 (5)	−0.0039 (4)	0.0016 (4)	0.0055 (4)
Cl3	0.0445 (7)	0.0638 (8)	0.0329 (6)	−0.0136 (6)	0.0039 (5)	−0.0157 (6)
La1	0.01471 (11)	0.01412 (11)	0.01787 (12)	0.00034 (9)	0.00007 (8)	−0.00003 (9)
Cl1	0.0181 (4)	0.0217 (5)	0.0371 (5)	0.0024 (4)	−0.0034 (4)	−0.0033 (4)

### Geometric parameters (Å, º)

**Table:**

C1—H1A	0.9900	C11—N11	1.458 (7)
C1—H1B	0.9900	C12—H12A	0.9900
C1—C2	1.504 (5)	C12—H12B	0.9900
C1—N1	1.472 (5)	C12—N12	1.451 (6)
C2—H2A	0.9900	N1—H1C	0.9100
C2—H2B	0.9900	N1—H1D	0.9100
C2—N2	1.465 (4)	N1—La1	2.794 (3)
C3—H3A	0.9900	N2—H2C	0.9100
C3—H3B	0.9900	N2—H2D	0.9100
C3—C4	1.495 (6)	N2—La1	2.754 (3)
C3—N3	1.470 (5)	N3—H3C	0.9100
C4—H4A	0.9900	N3—H3D	0.9100
C4—H4B	0.9900	N3—La1	2.748 (3)
C4—N4	1.479 (5)	N4—H4C	0.9100
C5—H5A	0.9900	N4—H4D	0.9100
C5—H5B	0.9900	N4—La1	2.876 (3)
C5—C6	1.500 (5)	N5—H5C	0.9100
C5—N5	1.481 (5)	N5—H5D	0.9100
C6—H6A	0.9900	N5—La1	2.863 (3)
C6—H6B	0.9900	N6—H6C	0.9100
C6—N6	1.490 (5)	N6—H6D	0.9100
C7—H7A	0.9900	N6—La1	2.756 (3)
C7—H7B	0.9900	N7—H7C	0.9100
C7—C8	1.509 (5)	N7—H7D	0.9100
C7—N7	1.474 (4)	N7—La1	2.731 (3)
C8—H8A	0.9900	N8—H8C	0.9100
C8—H8B	0.9900	N8—H8D	0.9100
C8—N8	1.473 (5)	N8—La1	2.715 (3)
C9—H9A	0.9900	N9—H9C	0.9100
C9—H9B	0.9900	N9—H9D	0.9100
C9—C10	1.498 (5)	N9—La1	2.766 (3)
C9—N9	1.485 (4)	N10—H10C	0.9100
C10—H10A	0.9900	N10—H10D	0.9100
C10—H10B	0.9900	N10—La1	2.722 (3)
C10—N10	1.469 (4)	N11—H11C	0.94 (5)
C11—H11A	0.9900	N11—H11D	1.00 (5)
C11—H11B	0.9900	N12—H12C	0.899 (19)
C11—C12	1.498 (7)	N12—H12D	0.915 (19)
			
H1A—C1—H1B	108.2	C4—N4—La1	115.4 (2)
C2—C1—H1A	109.8	H4C—N4—H4D	107.5
C2—C1—H1B	109.8	La1—N4—H4C	108.4
N1—C1—H1A	109.8	La1—N4—H4D	108.4
N1—C1—H1B	109.8	C5—N5—H5C	108.3
N1—C1—C2	109.5 (3)	C5—N5—H5D	108.3
C1—C2—H2A	109.8	C5—N5—La1	115.9 (2)
C1—C2—H2B	109.8	H5C—N5—H5D	107.4
H2A—C2—H2B	108.3	La1—N5—H5C	108.3
N2—C2—C1	109.3 (3)	La1—N5—H5D	108.3
N2—C2—H2A	109.8	C6—N6—H6C	108.5
N2—C2—H2B	109.8	C6—N6—H6D	108.5
H3A—C3—H3B	108.3	C6—N6—La1	115.0 (2)
C4—C3—H3A	110.0	H6C—N6—H6D	107.5
C4—C3—H3B	110.0	La1—N6—H6C	108.5
N3—C3—H3A	110.0	La1—N6—H6D	108.5
N3—C3—H3B	110.0	C7—N7—H7C	108.7
N3—C3—C4	108.6 (3)	C7—N7—H7D	108.7
C3—C4—H4A	109.7	C7—N7—La1	114.3 (2)
C3—C4—H4B	109.7	H7C—N7—H7D	107.6
H4A—C4—H4B	108.2	La1—N7—H7C	108.7
N4—C4—C3	109.6 (3)	La1—N7—H7D	108.7
N4—C4—H4A	109.7	C8—N8—H8C	107.7
N4—C4—H4B	109.7	C8—N8—H8D	107.7
H5A—C5—H5B	108.0	C8—N8—La1	118.3 (2)
C6—C5—H5A	109.3	H8C—N8—H8D	107.1
C6—C5—H5B	109.3	La1—N8—H8C	107.7
N5—C5—H5A	109.3	La1—N8—H8D	107.7
N5—C5—H5B	109.3	C9—N9—H9C	108.1
N5—C5—C6	111.5 (3)	C9—N9—H9D	108.1
C5—C6—H6A	109.8	C9—N9—La1	116.8 (2)
C5—C6—H6B	109.8	H9C—N9—H9D	107.3
H6A—C6—H6B	108.2	La1—N9—H9C	108.1
N6—C6—C5	109.6 (3)	La1—N9—H9D	108.1
N6—C6—H6A	109.8	C10—N10—H10C	108.4
N6—C6—H6B	109.8	C10—N10—H10D	108.4
H7A—C7—H7B	108.2	C10—N10—La1	115.7 (2)
C8—C7—H7A	109.7	H10C—N10—H10D	107.4
C8—C7—H7B	109.7	La1—N10—H10C	108.4
N7—C7—H7A	109.7	La1—N10—H10D	108.4
N7—C7—H7B	109.7	C11—N11—H11C	103 (3)
N7—C7—C8	109.7 (3)	C11—N11—H11D	119 (3)
C7—C8—H8A	109.6	H11C—N11—H11D	103 (4)
C7—C8—H8B	109.6	C12—N12—H12C	106 (4)
H8A—C8—H8B	108.2	C12—N12—H12D	106 (3)
N8—C8—C7	110.1 (3)	H12C—N12—H12D	107 (5)
N8—C8—H8A	109.6	N1—La1—N4	104.45 (9)
N8—C8—H8B	109.6	N1—La1—N5	67.35 (9)
H9A—C9—H9B	108.2	N2—La1—N1	61.58 (8)
C10—C9—H9A	109.8	N2—La1—N4	66.21 (9)
C10—C9—H9B	109.8	N2—La1—N5	105.61 (9)
N9—C9—H9A	109.8	N2—La1—N6	138.76 (10)
N9—C9—H9B	109.8	N2—La1—N9	69.13 (9)
N9—C9—C10	109.5 (3)	N3—La1—N1	68.85 (9)
C9—C10—H10A	109.9	N3—La1—N2	89.86 (9)
C9—C10—H10B	109.9	N3—La1—N4	60.42 (9)
H10A—C10—H10B	108.3	N3—La1—N5	117.46 (9)
N10—C10—C9	108.9 (3)	N3—La1—N6	67.65 (9)
N10—C10—H10A	109.9	N3—La1—N9	157.71 (9)
N10—C10—H10B	109.9	N5—La1—N4	170.99 (9)
H11A—C11—H11B	107.3	N6—La1—N1	77.84 (9)
C12—C11—H11A	108.0	N6—La1—N4	121.97 (9)
C12—C11—H11B	108.0	N6—La1—N5	61.26 (9)
N11—C11—H11A	108.0	N6—La1—N9	123.74 (9)
N11—C11—H11B	108.0	N7—La1—N1	134.62 (9)
N11—C11—C12	117.1 (5)	N7—La1—N2	141.70 (9)
C11—C12—H12A	109.5	N7—La1—N3	127.29 (9)
C11—C12—H12B	109.5	N7—La1—N4	120.46 (9)
H12A—C12—H12B	108.1	N7—La1—N5	68.18 (9)
N12—C12—C11	110.7 (5)	N7—La1—N6	73.46 (9)
N12—C12—H12A	109.5	N7—La1—N9	74.78 (9)
N12—C12—H12B	109.5	N8—La1—N1	139.36 (8)
C1—N1—H1C	108.2	N8—La1—N2	133.73 (9)
C1—N1—H1D	108.2	N8—La1—N3	73.49 (9)
C1—N1—La1	116.4 (2)	N8—La1—N4	68.06 (9)
H1C—N1—H1D	107.3	N8—La1—N5	120.47 (9)
La1—N1—H1C	108.2	N8—La1—N6	74.19 (9)
La1—N1—H1D	108.2	N8—La1—N7	62.48 (9)
C2—N2—H2C	108.7	N8—La1—N9	126.37 (8)
C2—N2—H2D	108.7	N8—La1—N10	76.02 (9)
C2—N2—La1	114.2 (2)	N9—La1—N1	93.69 (9)
H2C—N2—H2D	107.6	N9—La1—N4	114.06 (8)
La1—N2—H2C	108.7	N9—La1—N5	64.21 (8)
La1—N2—H2D	108.7	N10—La1—N1	138.45 (9)
C3—N3—H3C	108.3	N10—La1—N2	77.87 (9)
C3—N3—H3D	108.3	N10—La1—N3	122.62 (9)
C3—N3—La1	116.0 (2)	N10—La1—N4	63.40 (9)
H3C—N3—H3D	107.4	N10—La1—N5	119.86 (9)
La1—N3—H3C	108.3	N10—La1—N6	143.34 (10)
La1—N3—H3D	108.3	N10—La1—N7	74.08 (9)
C4—N4—H4C	108.4	N10—La1—N9	61.72 (8)
C4—N4—H4D	108.4		
			
C1—C2—N2—La1	53.4 (3)	C9—C10—N10—La1	51.9 (3)
C2—C1—N1—La1	38.3 (4)	C10—C9—N9—La1	37.7 (4)
C3—C4—N4—La1	−37.3 (4)	N1—C1—C2—N2	−60.2 (4)
C4—C3—N3—La1	−55.8 (4)	N3—C3—C4—N4	60.3 (4)
C5—C6—N6—La1	54.5 (4)	N5—C5—C6—N6	−56.6 (4)
C6—C5—N5—La1	32.3 (4)	N7—C7—C8—N8	−55.7 (4)
C7—C8—N8—La1	35.5 (4)	N9—C9—C10—N10	−57.8 (4)
C8—C7—N7—La1	50.1 (3)	N11—C11—C12—N12	−62.1 (7)

### Hydrogen-bond geometry (Å, º)

**Table:**

*D*—H···*A*	*D*—H	H···*A*	*D*···*A*	*D*—H···*A*
N1—H1*C*···Cl2^i^	0.91	2.53	3.405 (3)	160
N2—H2*C*···N11	0.91	2.40	3.284 (5)	164
N2—H2*D*···Cl2^ii^	0.91	2.72	3.417 (3)	134
N3—H3*D*···Cl2	0.91	2.59	3.497 (3)	176
N4—H4*D*···N11	0.91	2.49	3.267 (5)	144
N5—H5*C*···Cl2^i^	0.91	2.74	3.573 (3)	153
N7—H7*C*···Cl3^iii^	0.91	2.54	3.376 (3)	154
N7—H7*D*···Cl3	0.91	2.63	3.470 (3)	154
N8—H8*D*···Cl2	0.91	2.40	3.292 (3)	168
N10—H10*C*···Cl2^ii^	0.91	2.57	3.445 (3)	161
N11—H11*D*···Cl2^ii^	1.00 (5)	2.83 (5)	3.642 (5)	139 (4)

Symmetry codes: (i) *x*−1, *y*, *z*; (ii) −*x*+3/2, *y*−1/2, −*z*+1/2; (iii) −*x*+1, −*y*+1, −*z*+1.
